# Effects of Counter Anions on AC and DC Electrical Conductivity in Poly(Dimethylsiloxane) Crosslinked by Metal-Ligand Coordination

**DOI:** 10.3390/polym13060956

**Published:** 2021-03-20

**Authors:** Angelika Wrzesińska, Aleksandra Wypych-Puszkarz, Izabela Bobowska, Jacek Ulański

**Affiliations:** Department of Molecular Physics, Faculty of Chemistry, Lodz University of Technology, Zeromskiego 116, 90-924 Lodz, Poland; angelika.wrzesinska@edu.p.lodz.pl (A.W.); izabela.bobowska@p.lodz.pl (I.B.)

**Keywords:** PMDS metal–ligand complexes, conduction mechanism, broadband dielectric spectroscopy

## Abstract

There is an urgent need for the development of elastic dielectric materials for flexible organic field effect transistors (OFETs). In this work, detailed analysis of the AC and DC electrical conductivity of a series of flexible poly(dimethylsiloxane) (PDMS) polymers crosslinked by metal-ligand coordination in comparison to neat PDMS was performed for the first time by means of broadband dielectric spectroscopy. The ligand was 2,2-bipyridine-4,4-dicarboxylic amide, and Ni^2+^, Mn^2+^, and Zn^2+^ were introduced for Cl^−^, Br^−^, and I^−^ salts. Introduction of metal salt and creation of coordination bonds resulted in higher permittivity values increasing in an order: neat PDMS < Ni^2+^ < Mn^2+^ < Zn^2+^; accompanied by conductivity values of the materials increasing in an order: neat PDMS < Cl^−^ < I^−^ < Br^−^. Conductivity relaxation time plot as a function of temperature, showed Vogel-Fulcher–Tammann dependance for the Br^−^ salts and Arrhenius type for the Cl^−^ and I^−^ salts. Performed study revealed that double-edged challenge can be obtained, i.e., dielectric materials with elevated value of dielectric permittivity without deterioration too much the non-conductive nature of the polymer. This opens up new perspectives for the production of flexible dielectrics suitable for gate insulators in OFETs. Among the synthesized organometallic materials, those with chlorides salts are the most promising for such applications.

## 1. Introduction

The fast-growing market of organic electronics requires constant development of new materials. Elaboration of a fully stretchable dielectric material for applications in flexible electronics is one of the main challenges in emerging organic electronics technology. Fabrication of highly stretchable organic field-effect transistors (OFETs) and flexible capacitors, which are essential elements of the electronic devices, would open a path for the creation of new appliances for in medicine, communication, and entertainment, thereby improving the standard of human life.

In design of flexible OFETs, special attention is paid to both mechanical and electrical properties of the semiconductor layer, and to the properties of the dielectric layer. In the case of the latter, a lot of attention has been paid to the synthesis of materials which could replace the SiO_2_ gate dielectric [1]. Commonly used Si-based electronics are susceptible to scratching, rupturing, and other damage coming from bending or stretching, leading to functionality losses. On the other hand, elastic polymer dielectrics are characterized by overly low dielectric permittivity (**k**) and are not able to create sufficient charge carrier density in the OFET channel. Hence, there is a great demand for dielectric materials with low roughness and high elasticity that possess **k** values higher than polymers.

Nowadays, the challenge is to synthesize new materials which will fulfill strict requirements for elastic electronics [1]. The dielectric permittivity of an elastomer can be improved by adding various inorganic fillers to the polymer matrix [2,3]. Usually, these composites are characterized by worst mechanical and morphological properties compared to a neat polymer. In comparison to polymeric materials, inorganic–organic dielectric materials usually exhibit lower stretchability and higher roughness, causing obstacles for their application in flexible electronics [4]. However, our last findings evidenced that the addition of a star-shaped copolymer with a small amount of TiO_2_ to the PMMA matrix significantly decreased the roughness of this dielectric layer from 15.3 to 0.43 nm and improved the efficiency of the obtained OFETs considerably [5].

Chemical modification of elastomers by introducing polar groups was reported to be an effective way of increasing their dielectric properties [6,7]. Nevertheless, this approach leads to large increases in the glass transition temperatures (*T*_g_) of the resulting materials, making them brittle [3,6]. An alternative approach for increasing the complex dielectric permittivity of a polymer without an undesirable increase in its stiffness is based on the introduction of a dynamic non-covalent metal-ligand coordination bond into the polymer matrix [8,9,10]. Since 2011, when the first paper on self-healing polymers based on metal-ligand interactions was publish by the group of Weder [11], great progress in these materials has been achieved [12]. Plenty of options exist in designing polymers crosslinked by metal-ligand coordination with advanced functionalities. There are possible applications for various PMDS crosslinked by metal-ligand coordination in other technological areas, e.g., as charge transfer mediators for biosensors, or as artificial muscle components having the ability to restore a high dielectric strength after recovery from mechanical damage [13].

In our previous work [10], we reported syntheses of a series of metallopolymers based on polydimethylsiloxane (PDMS) crosslinked by metal (Ni^2+^, Mn^2+^, Zn^2+^)–bipyridine (bpy) coordination with Cl^−^ as a counter anion and investigated their dielectric properties and molecular dynamics. These materials have almost two times higher dielectric permittivity than neat PDMS. The nature of the cation used directly affected the dielectric properties of the materials obtained due to the dipolar nature of the coordination bonds [13,14]. Nevertheless, this approach requires the introduction of metal cations into the polymer systems in the form of metal salts, for example, halides, tetrafluoroborates, or perchlorates [8,10,15]. For this reason, free remnant counter anions can noticeably affect the conductivity of such materials. Recently, Rao et al. reported prominent effects by the anion size and mobility on the leakage current of OFETs with metal−ligand coordinated PDMS used as the dielectric layer [8]. This could indicate that the challenge is double-edged, namely, increasing the dielectric permittivity of the organometallics without deterioration of the non-conductive nature of the polymer. Therefore, a detailed examination of the conductivity in such metallopolymer systems is desired to understand the charge transport mechanisms.

Ion conductivity can be studied using broadband dielectric spectroscopy (BDS), which allows measurements in a wide range of frequencies and temperatures [16]. Additionally, measurements conducted by BDS allow one to examine the conductivity relaxation processes in systems [16,17]. In this work, we examined the alternating current (AC) and direct current (DC) electrical conductivity of PDMS coordinated by metal−ligand bonds using three different metal cations (Ni^2+^, Mn^2+^, and Zn^2+^) and halide counter anions (Cl^−^, Br^−^, and I^−^) by means of BDS. To the best of our knowledge, this is the first attempt to analyze the influences of counter anions on temperature-dependent conductivities and to create an activation map of conductivity relaxation time in organometallic materials.

## 2. Materials and Methods

**Figure 1:**
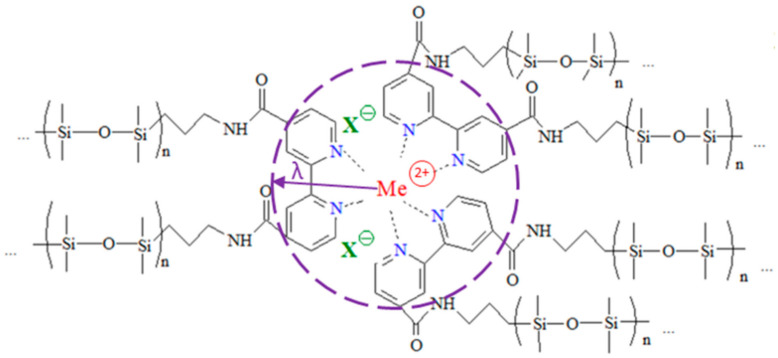
Molecular structure of bpyPDMS-MeX_2_ investigated in this work (where Me^2+^: Ni^2+^, Mn^2+^, or Zn^2+^; and X^−^: Cl^−^, Br^−^ or I^−^). Dashed line presents size of the Columbic cage, where λ corresponds to its average size.

In this work, we used PDMS crosslinked through metal–bpy coordination with nine different crosslinking salts (bpyPDMS-MeX_2_, where Me: Ni^2+^, Mn^2+^, or Zn^2+^; and X: Cl^−^, Br^−^ or I^−^). Their chemical structures are presented in Figure 1. All complexes were synthesized from detailed descriptions of their synthesis reported elsewhere [10].

### 2.1. Differential Scanning Calorimetry (DSC)

DSC curves were measured using a DSC 3 calorimeter from Mettler Toledo (Columbus, OH, USA). Thermograms were obtained at a scan rate of 10 K/min in a temperature range from 133 to 373 K with a 2 mL/min nitrogen gas purge in standard, sealed aluminum crucibles. The temperature and heat flow were calibrated using indium and zinc melting point standards. Values of *T*_g_ were determined from the second run as a midpoints of respective glass transition step by STAR^e^ Evaluation Software (Columbus, OH, USA). BpyPDMS-MeX_2_ was dissolved in tetrahydrofuran (THF) solvent, then poured layer by layer in crucibles. After each layer, we waited till THF evaporated on the hot plate at 323 K. Before measurements in crucibles with around 10 mg of product, solvents were evaporated from the samples by keeping them in an oven overnight under reduced pressure at temperature of 323 K.

### 2.2. Broadband Dielectric Spectroscopy (BDS)

BDS measurements were performed by a Novocontrol^®^ GmbH Concept 80 Broadband Dielectric Spectrometer (Montabaur, Germany). All samples were measured within a frequency range from 10^−1^ to 10^6^ Hz. The measurement temperature was controlled by a Quatro Cryosystem (Montabaur, Germany) of a BDS setup in a range from 143 to 373 K with a stability better than 0.5 K. All investigated systems were prepared for dielectric spectroscopy measurements by sandwiching the specimen between two gold-plated flat electrodes with dimensions of 20 (top) by 30 mm (bottom), respectively. The electrodes were separated from each other by two 100 μm-thick glass wires parallel to each other. PDMS was casted as received between gold-plated electrodes, whereas organometallics materials were dissolved in THF (because of their solidity at room temperature). Dissolved complexes were poured out on the electrode between the glass layer by layer up to a thickness equal to that of the glass wires. After each layer, we waited till THF evaporated on the hot plate at 323 K. Before measurements, upper electrodes were put on cast samples and sandwiched arrangements were made. Such prepared samples were kept under load in a vacuum dryer overnight in reduced pressure and at a temperature of 323 K.

## 3. Results and Discussion

### 3.1. Differential Scanning Calorimetry

**Figure 2:**
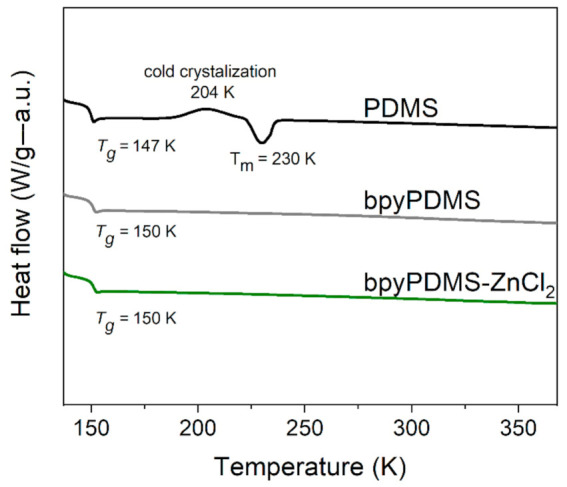
DSC thermograms of PDMS, bpyPDMS, and exemplary organometallic compound bpyPDMS-ZnCl_2_ (thermograms are vertically shifted for better visualization).

Figure 2 presents DSC thermograms for neat PDMS, bpyPDMS, and the exemplary bpyPDMS-ZnCl_2_ metalloorganic complex. In the case of neat PDMS, a characteristic step typical for glass transition was observed at 147 K (as a midpoint). Other two processes detected at higher temperatures are connected with the crystalline phase. One of them is located at around 204 K, has an exothermic nature, and corresponds to the process of cold crystallization. The second one appears as a more pronounced exothermic peak at about 230 K, revealing melting of the crystalline PDMS phase. In the case of bpyPDMS and other organometallic complexes, one transition at about 150 K was observed and assigned to the *T_g_* of investigated materials. These measurements evidenced that incorporation of bpy moieties suppressed the crystallization process of PDMS and led to the creation of fully amorphous systems (see Figure 2) [10]. It is important to underline that the structures of all investigated complexes were amorphous, and similar thermograms were detected for all organometallic complexes.

### 3.2. Broadband Dielectric Spectroscopy

#### 3.2.1. Isothermal Representations of the Dielectric Responses of the Studied Systems

**Figure 3:**
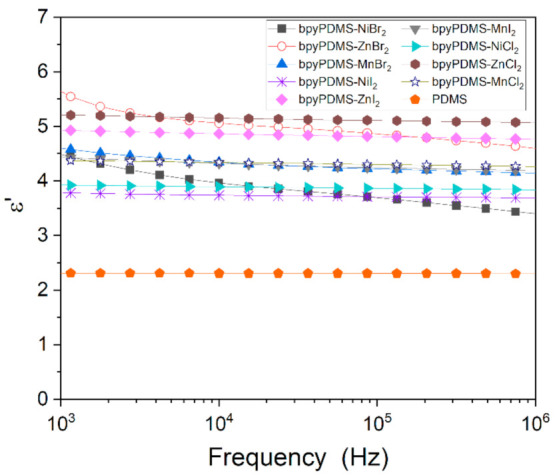
Frequency dependence of the real part of dielectric permittivity ε′ at 293 K for PDMS and organometallic compounds.

Studies of frequency dependence of the real part of dielectric permittivity ε′ at 293 K of PDMS and metalloorganic complexes are shown at Figure 3. All samples exhibited a flat response over a broad frequency range. It can be observed that metalorganic materials are characterized by elevated values of dielectric permittivity as compared with neat PDMS. Such behavior is caused by the dipolar metal-ligand coordination bonds in PDMS materials crosslinked by metal-ligand coordination [13,14]. All bpyPDMS-MeX_2_ systems displayed elevated dielectric permittivity, depending on the type of cation originating from the metal salt; the dielectric permittivity increased in this order: neat PDMS < Ni^2+^ < Mn^2+^ < Zn^2+^. The highest dielectric permittivity, almost twice as high as for neat PDMS, was observed for bpyPDMS-ZnCl_2_, which is in accordance with our previous findings [10].

#### 3.2.2. Descriptions of DC and AC Electrical Conductivity Contributions in the Complex Conductivity of the Studied Systems

**Figure 4:**
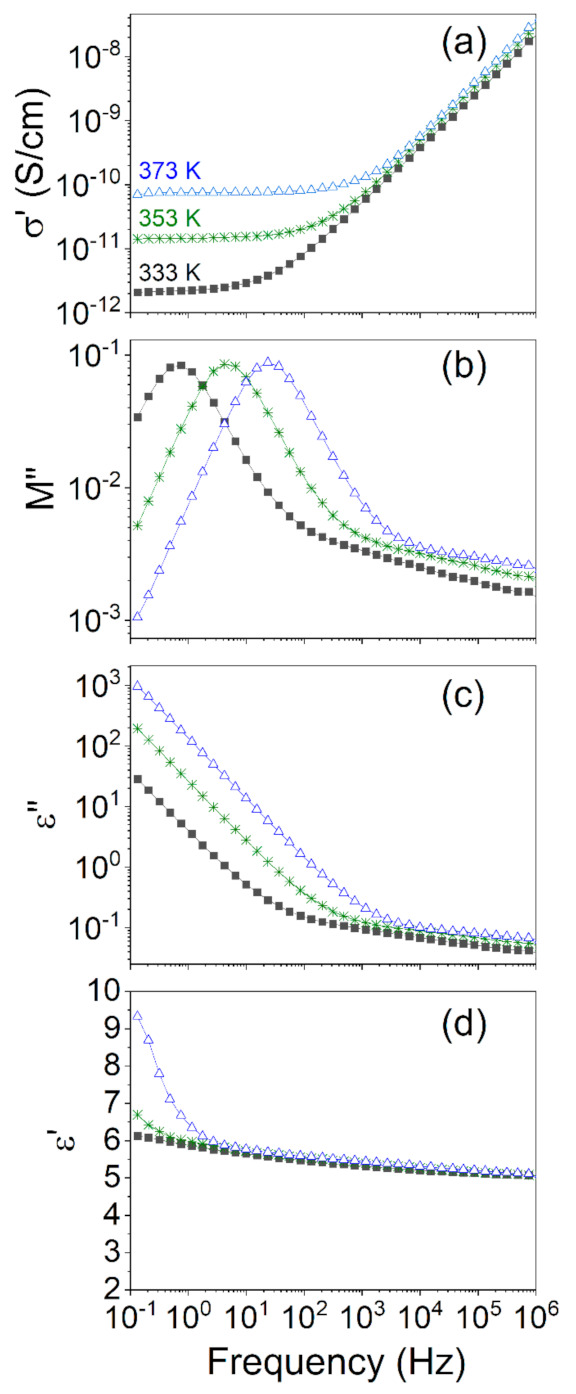
Different representations of the BDS data recorded for bpyPDMS-ZnCl_2_ at a few temperatures 333, 353, and 373 K namely, (**a**) the real part of the conductivity (σ’), (**b**) the imaginary part of the modulus (M”), (**c**) the imaginary part of the dielectric permittivity (ε”), and (**d**) the real part of the dielectric permittivity (ε’) vs. frequency.

Variations of the real part of conductivity as a function of frequency at various temperatures for the chosen metalorganic complex are given in Figure 4a. By analyzing this figure, it can be seen that conductivity increases with both frequency and temperature. One can deduce that the conduction mechanism is thermally activated. However, with increasing frequency, conductivity starts to be less and less temperature dependent. As proposed by von Hippel [18], the alternating current conductivity (σ_ac_) can be described as the total sum of all energy dissipative effects, which also include actual ohmic conductivity caused by drifting charge carriers and frequency dielectric dispersions. With decreasing frequency, charge carriers must overcome larger distances. Moreover, their transport is greatly limited by the presence of isolated conductive spots. It is a well-known fact [19,20] that the dispersion of AC conductivity for heterogenous and disordered solids increases with frequency. The fact that AC conductivity of different types of disordered solids possesses a similar type of frequency dependence is very interesting and still not fully understood [19,21,22]. Herein it can be underlined that besides BDS, cycling voltammetry can be used to study conductivity phenomena in different types of materials [23]. The difference between the AC and DC contributions to complex conductivity in the studied systems will be discussed in the following subsections.

Contrary to this, DC conductivity is connected with the concentration and mobility of charge carriers; therefore, its contribution is facilitated and reinforced by cooperative movements of the polymer units or chains, which are characteristic of a viscoelastic or melted polymer state. The acceleration of polymer dynamics with temperature results in enlargement of the Coulombic cage [16,24] and increases the possibility of counter anion skipping. 

The observed tendency of the conductivity vs. frequency relationship is also supported by the AC universality law [21,25]:

σ_ac_(ν) = σ_dc_ + A(ν)^s^ (1)

where σ_dc_ is the frequency limiting value of σ_ac_(ν) when ν ≥ 0, and A and s are parameters depending on temperature and type of material.

At lower frequencies, conductivity is determined only by the migration of electric charges and reaches a certain constant value, called a plateau value, which is representative of σ_dc_ (Figure 4a). Based on the experimental data, it is possible to predict the frequency at which the DC starts to dominate over AC’s contribution [26]. The increase in the signal in the loss part representation of dielectric permittivity (see Figure 4c) is due to the increase of DC’s conductivity contribution with increasing temperature. In the measured high-frequency range, dipole relaxations dominate in the dielectric response of the material. The beginning of the frequency-dependent conductivity corresponds to the characteristic time τ_c_:

τ_c_ = 1/(2πν*) (2)

where ν* is crossover frequency [27].

According to the theory of linear conductivity response, (τ) > 1/(2πν*), assuming that the movement of ions in thermal equilibrium is diffusive and its mean square shift <λ^2^ (t)> increases linearly with time. Above the crossover frequency, i.e., when ν > ν *, σ′(ν) representation increases with frequency, and (τ) < 1/(2πν*). In this case, the movement of ions exhibits a sub-diffusive mechanism; however, their mean square shift also increases linearly with time, as in the case of diffusive ions. It should also be noted that the starting frequency for the dispersion part of the conductivity is thermally activated and this activation energy is identical to the activation energy of DC conductivity σ_dc_ [27].

In Figure 4 and Supplementary Materials Figure S1 there are different representations of the BDS data recorded for bpyPDMS-ZnCl_2_ and bpyPDMS-NiBr_2_, respectively, which are compounds selected from among the studied metalloorganic complexes. Therein, we compare different dielectric representations, namely, σ’, M”, ε”, and ε’, and analyze how different conductivity contributions influence their values. By analyzing conductivity spectra in Figure 4a it is possible to distinguish two regimes. The first was assigned to AC conductivity, while ions moved in a limited space. The second one is the DC conductivity (σ_0_), which is attributed to the ionic drift. In line with theoretical expectations, DC conductivity is observed for low and medium frequencies, whereas AC conductivity has a dominant contribution at the high-frequency region. Changes observed in conductivity representations upon cooling (Figure 4a) are connected with slowing down of ions’ mobility which results in a decrease of the DC conductivity plateau. In Figure 4b, the loss part of the electric modulus is shown. A representation of the dielectric data in the form of the complex electric modulus M*(ν) = M′(ν) + iM″(ν), defined as the inverse of complex relative permittivity M*(ν) = 1/ε*(ν), is another significant powerful tool for analyzing the electric behavior of polymers crosslinked by metal-ligand coordination. The crossover frequency region of AC and DC conductivity is reflected as the maximum in the M” representation against frequency (Figure 4b). In Figure 4c, the contribution of DC conductivity in ε” representation is shown as a straight line with a slope of approximately −1. Contrary to the above-mentioned representations of the real part of dielectric permittivity, it is almost insensitive to conductivity changes (Figure 3). The rapid increase in ε’ observed in Figure 4d in the low-frequency range and a high temperature, i.e., 373 K, resulted from the electrode polarization.

#### 3.2.3. Comparison of Conductivity in Neat PDMS and Its Metalloorganic Complexes

**Figure 5:**
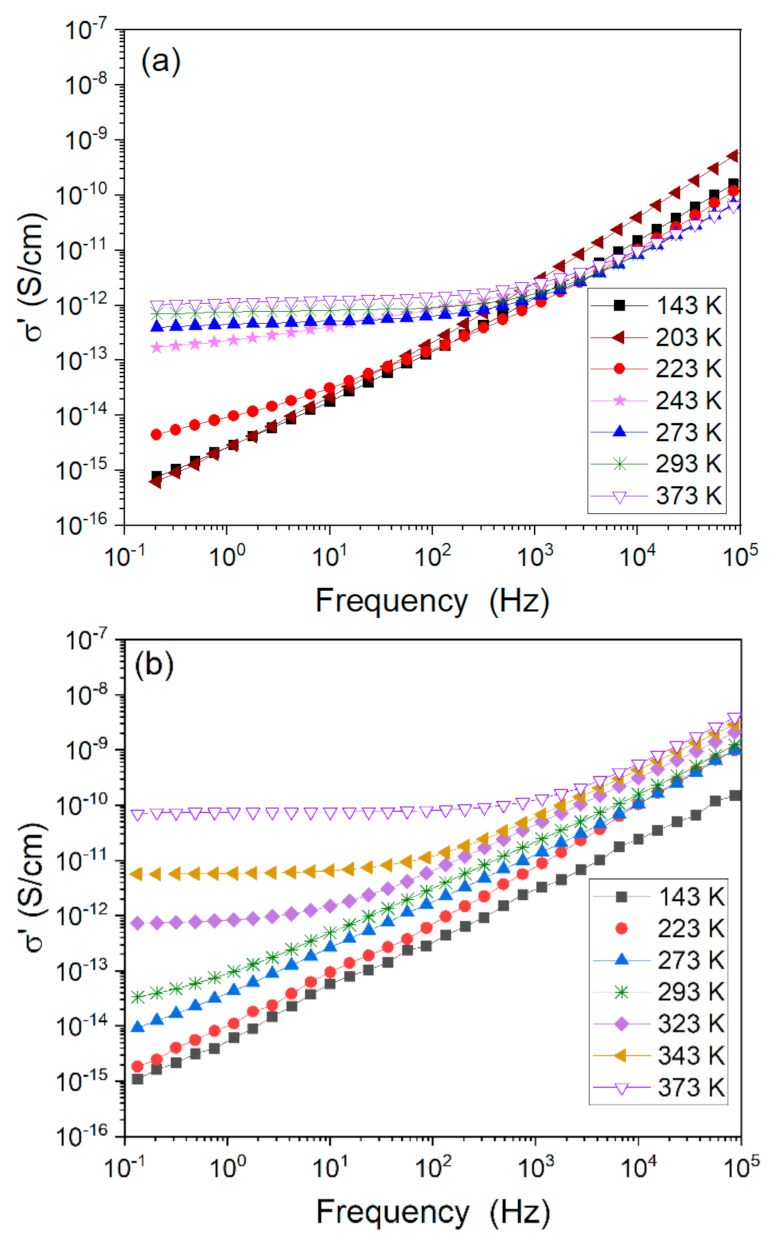
Representation of the real part of conductivity (σ’) at selected temperatures for (**a**) neat PDMS and (**b**) bpyPDMS-ZnCl_2_ as a function of frequency.

Analyzing dielectric data can provide valuable information regarding the electrical properties–structure relationship. Figure 5 depicts the real part of conductivity (σ’) vs. frequency at a few selected temperatures for neat PDMS (Figure 5a) and for PDMS-based metal–ligand compounds with the example of bpyPDMS-ZnCl_2_ (Figure 5b). That particular crosslinked system exhibited higher conductivity than neat PDMS. This phenomenon was expected for all examined metal-ligand coordination systems and is caused by mobile anions (originated from salts used for crosslinking procedure), which are present in those materials.

In Figure 5a,b it is visible that below *T_g_* (T lower than 153 K), the DC conductivity plateau is very short or even not observable, and the conductivity is much lower because ionic conductivity is very impeded in a glassy state. When comparing the DC conductivity of neat PDMS and its metalloorganic complex, one should remember that the chemical compositions of these materials are very different. Accordingly, DC conductivity in neat PDMS arises from the remnant ions of synthesis or impurities, whereas in organometallic compounds it is driven by the introduced counter ions. Further analysis showed that at a certain frequency the mechanism of conductivity of studied materials is changed and conductivity tends toward constant values, approaching the DC conductivity limit at low frequencies. This frequency is seen in Figure 5a,b as a point of the change in the slope of each curve. The frequency range of a plateau extends with increasing temperature. However, it differed for all investigated samples. In the case of neat PDMS, conductivity changed ca. an order of magnitude with a temperature increase from 223 to 373 K (see Figure 5a). For crosslinked metal–ligand complexes, this change was up to five orders of magnitude in the same temperature range (see Figure 5b). The increase of conductivity in σ’(ν) representation after the DC conductivity plateau region is attributed to the beginning of the so-called sub-diffusive regime [28]. At high frequencies, charge carriers explore the energy landscape of the disordered environment in a sub-diffusive way. Only fully diffusive movements give rise to the DC conductivity plateau [29]. Summarizing, the change of conductivity value and its temperature behavior for organometallic materials and PDMS are in a large way caused by incorporation of halide counter anions. Nevertheless, it is important to point out that neat PDMS and its organometallic complexes exhibit purely capacitative responses in the investigated temperature range. The representation of the phase angle for the studied samples is equal to or oscillates around a value equal to −90°, which confirms the former statement (see Figure S2) [30]. 

#### 3.2.4. Influences of Counter Anions on DC Conductivity in Metalloorganic Complexes

**Figure 6:**
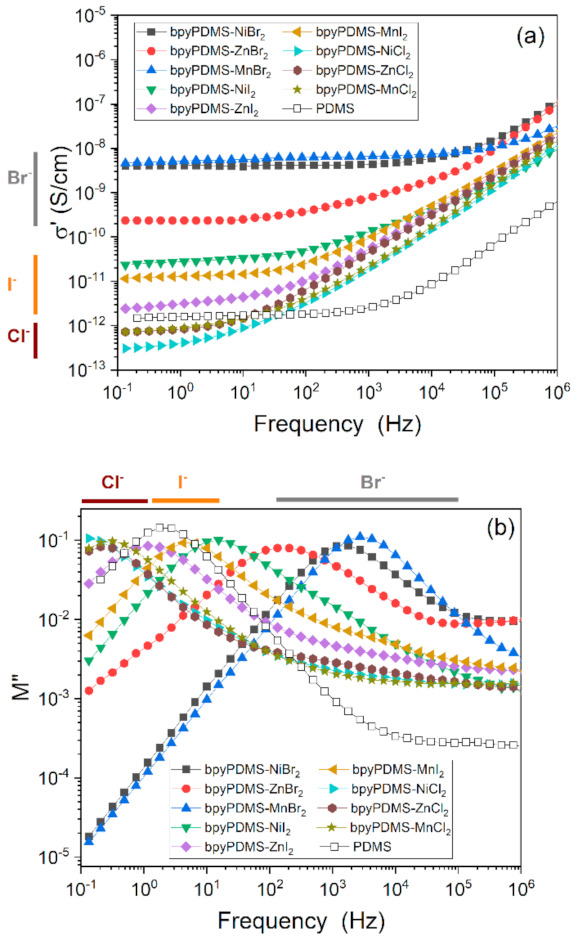
(**a**) Real part of conductivity (σ’) and (**b**) imaginary part of modulus (M”) plots of PDMS and all investigated organometallic compounds vs. frequency at 323 K.

The BDS spectra at 323 K for all investigated organometallic samples and neat PDMS are gathered in Figure 6a,b. The difference in behavior between the studied materials, for both the real part of conductivity σ’(ν) (Figure 4a) and the imaginary part of modulus M”(ν) (loss modulus) (Figure 4b), was discussed in the previous subsection. Herein we compare the isothermal responses of investigated samples at 323 K. This value corresponds to the conductivity relaxation attributed to the reorganization of counter ions in the metal–ligand crosslinked PDMS systems [16].

For the materials investigated, analysis of the peak maximum in the imaginary part of the electric modulus M″(ν) (Figure 4b and Figure 6b) is essential. Visible maxima in M″(ν) representation correspond roughly with the frequency points at which the conductivity transferred from mostly AC to mostly DC (Figure 4a and Figure 6a). The change of conduction mechanism is connected with the molecular structure of the investigated system and the previously mentioned size of the Coulombic cage (Section 3.2.2). In organometallic compounds, the counter anions are located in the close surroundings of the metal cations, and create the coordination bonding; bipyridine molecules (see Figure 1) have mutual interactions with metal cations. AC conductivity is attributed to fast and short-distanced movements inside the Coulombic cage (high frequency region in Figure 6a), whereas DC is due to mobility of counter ions over longer distances (lower frequency region in Figure 6a), during which ions will overcome larger energy barriers in the system. This can be understood as an escaping of ions from the Coulombic cage. 

Among the studied anions, chlorides had the lowest values of conductivity and the smallest DC conductivity contribution to total conductivity response as compared to other organometallic materials and neat PDMS, which can be seen by the short plateau region. This is a result of varying diffusion properties depending on the size and electronegativity of the anion used (see Figure 6a). The recorded DC conductivity for chlorides ranged from 3.0 × 10^−13^ to 7.2 × 10^−13^ S/cm, for iodides from 2.5 × 10^−12^ to 3.3 × 10^−11^ S/cm, and for bromides from 2.5 × 10^−10^ to 4.5 × 10^−9^ S/cm, whereas neat PDMS exhibited a plateau at 1.5 × 10^−12^ S/cm. The anions present in the investigated organometallic complexes can be arranged in terms of increasing radius in the following order: Cl^−^, Br^−^, and I^−^; and their electronegativity arranges them in the reverse order. In the investigated systems, the coordination interaction between metal ions and bipyridine molecules proceeded spontaneously due to changes of Gibbs free energy, and more specifically due to an increase of entropy, whereas halide anions remained unbounded. This was well described by Li et al., who analyzed coordination bonds in self-healing polymers and arranged the complexing groups by classification of Lewis acids and bases [12]. Accordingly, iodides are classified as soft bases due to their intermediate electronegativity and large size, whereas chlorides and bromides are placed in the borderline group between soft and hard bases due to their higher electronegativities and smaller sizes. These parameters determine the interactions of these counter anions with the surrounding environment and affect their diffusion coefficients and mobilities. Following this model, the DC conductivities in the investigated system should have increased in the order: chlorides, bromides, iodides. Herein, the order between bromides and iodides was exchanged. The organometallic compounds comprising bromides exhibited the highest DC conductivities among the investigated system. They were also characterized by the broadest frequency, i.e., the long DC plateau region in the real part of conductivity. This phenomenon is partially connected with the highest mobility being possessed by the bromides among the halide anions used [31]. Considering that the conductivity of the studied system is a function of both charge density and charge mobility, it can be concluded that the highest conductivity of complexes based on bromide salts can be explained by the high density of mobile counter anions, which was most likely due to the weak Coulombic interaction between bromides and complexing metal.

Conductivity, i.e., charge transport in materials, is frequency and temperature-dependent. In order to study the physical origin of this process, theoretical conduction models can be applied. The temperature-related evolution of conductivity (σ_0_) can be well described in the whole temperature range in terms of an Arrhenius-type relation (3) or by a Vogel–Fulcher–Tammann (VFT) expression (4):

(3) $$\sigma \left(T\right)={\;\sigma }_{0}\exp\left(\;-\;\frac{E_{A}}{k_{B}T}\right),$$

(4) $$\sigma \left(T\right)={\;\sigma }_{0}\exp\left(-\;\frac{{\mathrm{BT}}_{0}}{T-T_{0}}\right)$$

where σ_0_ is a pro-exponential factor, E_A_ is the activation energy, B is a constant, and T_0_ is the Vogel temperature.

**Figure 7:**
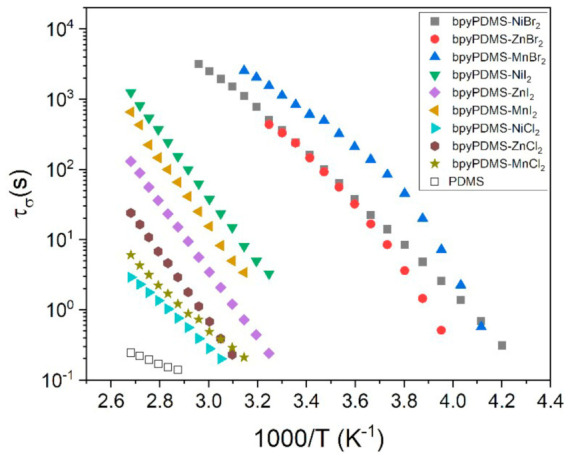
Conductivity relaxation time plot of PDMS and all investigated metalloorganic complexes as functions of 1000/T.

The activation map was obtained for the temperature interval 227–385 K, corresponding to the possible temperature range of applications of these materials. The visible maxima at the representation of M″(ν), which marks the beginning of the DC component, allowed us to create the relaxation map for neat PDMS and all measured organometallic complexes as a function of 1000/T; see Figure 7. On the basis of the conductivity relaxation time plot, different temperature regimes of the DC component occurrence can be observed. The map shows that the temperature of the occurrence of the DC component de pends on the counter anion used. The shapes of the obtained temperature dependences confirmed VFT type for bromides and Arrhenius type for the rest of the samples. 

One can also notice that among the investigated salts, bromides activated DC conductivity at the lowest temperature (~253 K), whereas chlorides and iodides did so at about 312 K that is almost 60 K higher. This may have been due to differences in diffusion coefficients and mobilities of the counter anions. This assumption is supported by computer simulations of anions mobility performed by Lee et al. [31]. They reported that the diffusion coefficient and mobility for Br^−^ are about 10% higher than the values calculated for chlorides or iodides. Hence, the anion mobility as a function of ionic radii of halides exhibits a distinct maximum in the case of bromides [31]. This fact may explain why the conductivity mechanism for organometallic compounds containing bromides is activated at a lower temperature as compared to other ions. The representation of the real part of the dielectric permittivity (ε’) for samples crosslinked by bromides shows an unexpected decrease at temperatures above 343 K. Such behavior suggests the breakdown of the coordination bonds, which become unstable at a certain temperature and then renew when the sample is cooled (see Figures S3 and S4). The representation of ε’ and σ’ vs. frequency at 293 K during temperature-controlled measurements and after them (samples were gradually cooled from 393 to 293 K) proved the reversibility of the crosslinking process in organometallic compounds with bromides.

For application reasons, the DC conductivity should exhibit the smallest possible value and should be activated at the highest possible temperatures, so samples containing chlorides as counter anions are expected to have the best dielectric properties, as they exhibited the lowest temperature-dependent conductivity value, similar to that of neat PDMS. The bromide ions activated the conduction mechanism earlier (at lower temperatures than chlorides and iodides), so they can potentially generate higher leakage current in organic electronic devices in comparison with other tested counter anions. The analysis of conductivity shows that organometallic complexes, especially those containing chlorides as counter anions, are better than commercial liquid PDMS because they exhibit solid state properties in a broad temperature range and activation of DC conductivity contributions at slightly higher temperatures, i.e., lower frequencies. This is in agreement with the findings of Rao et al., who investigated the organometallic complexes of Fe^2+^ and Zn^2+^ with various counter anions, such as Cl^−^, BF_4_^−^, ClO_4_^−^ and CF_3_SO_3_^−^ as dielectric layers in OFETs. Among them, complexes comprising chlorides had the best properties: hysteresis-free transfer characteristics and low values of gate leakage current. They attributed these properties to the strong columbic interaction between metal cations and the small Cl^−^ anions, which can prevent mobile anions from drifting under gate bias [8]. To summarize, the conductivity mechanism is determined by two parameters—the density of the carriers and their mobility—and usually, one of them is dominant. In the case of Br^−^ counter ions, they are easily released from the Coulomb cage; thus, the conductivity is controlled by the mobility of the ions in the polymer matrix. Metalorganic complexes with Br^−^ counter ions exhibited dependence for molecular relaxations above *T_g_* that can be described by the VFT equation. The remaining studied systems showed Arrhenius relationships, because counter ions are more strongly bound to the cations by Coulomb forces. In such a case, the number of mobile ions is much smaller as compared with a system with Br^−^ and the release of counter ions from the Coulomb cage is thermally activated according to the Arrhenius relationship.

## 4. Conclusions

In the present contribution we employed broadband dielectric spectroscopy to investigate the effects of halide counter anion type on the AC and DC electrical conductivity of poly(dimethylsiloxane) crosslinked by metal-ligand coordination. It was found that an elevated value of dielectric permittivity depends on the type of cation from the metal salt and increases in the order: neat PDMS < Ni^2+^ < Mn^2+^ < Zn^2+^. The conductivity values of the studied materials depending on the metal halide salts used for their synthesis can be arranged in the following decreasing order: Br^−^ > I^−^ > Cl^−^ > neat PDMS. The type of counter anion significantly influenced the DC conductivity. For bromide-based materials, the contribution of DC conductivity was visible already at ca. 253 K, whereas for chloride- and iodide-based materials above ca. 310 K. The conductivity relaxation map demonstrated Arrhenius-type dependencies for Cl^−^, I^−^, and neat PDMS, and VFT-type for Br^−^. Among the studied anions, chloride-based materials had the lowest values of conductivity and the smallest DC conductivity contribution to total conductivity response as compared to other organometallic materials and neat PDMS; that can be seen by the short plateau region. The recorded DC conductivity for organometallic complexes based on chlorides with various metal cations ranged from 3.0 × 10^−13^ S/cm to 7.2 × 10^−13^; for iodides it was from 2.5 × 10^−12^ to 3.3 × 10^−11^ S/cm; for bromides it was from 2.5 × 10^−10^ to 4.5 × 10^−9^ S/cm. Neat PDMS exhibited a plateau at 1.5 × 10^−12^ S/cm at 323 K. The low conductivity of chloride salt-based complexes can be explained by their low densities of mobile counter anions, which are most likely due to the strong Coulombic interactions between the chlorides and the complexing metal. These materials trade-off elevated dielectric permittivity with an only slight conductivity increase. For this reason, metal chlorides seem the most promising salts for the synthesis of elastic dielectrics for OFETs, since low values of gate leakage current are required.

## Data Availability

The data presented in this study are available on request from the corresponding author.

## Supplementary Materials

The following are available online at https://www.mdpi.com/2073-4360/13/6/956/s1. Figure S1: Different representations of the BDS data recorded for bpyPDMS-NiBr2 at a few temperatures: 263, 283, 303, and 323 K—namely, (a) the real part of the conductivity (σ’), (b) the imaginary part of the modulus (M”), (c) the imaginary part of the dielectric permittivity (ε”), and (d) the real part of the dielectric permittivity (ε’) vs. frequency. Figure S2: Frequency dependence of the real part of the dielectric permittivity for bpyPDMS-NiBr_2_ at 293 K (during measurement), at 293 K (after measurement), and at 393 K (during measurement). Figure S3: Frequency-temperature dependences of (a) the real part of the dielectric permittivity and (b) the real part of the conductivity (σ’) for the bpyPDMS-NiBr_2_ sample.
