# Supplementary material for: Effects of Counter Anions on AC and DC Electrical Conductivity in Poly(Dimethylsiloxane) Crosslinked by Metal-Ligand Coordination
Source: Polymers (Basel). 2021 Mar 20;13(6):956. doi: 10.3390/polym13060956 (PMC8003853; doi:10.3390/polym13060956)
Supplement: Supplementary file 1 [file polymers-13-00956-s001.pdf]

Supporting Information

Effects of Counter Anions on AC and DC Electrical Conductivity in Poly(Dimethylsiloxane) Crosslinked by Metal–Ligand Coordination

Angelika Wrzesińska, Aleksandra Wypych-Puszkarz*, Izabela Bobowska, and Jacek Ulański*

Department of Molecular Physics, Faculty of Chemistry, Lodz University of Technology, Zeromskiego 116, 90-924 Lodz, Poland

*Correspondence: jacek.ulanski@p.lodz.pl, aleksandra.wypych@p.lodz.pl , Tel.:+48 42 631 32 05

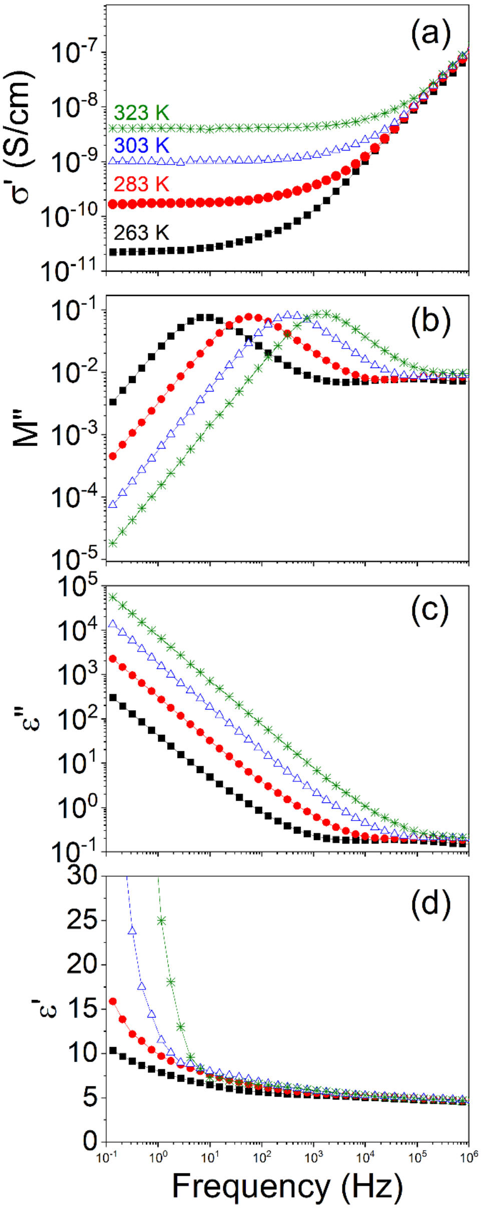

Figure S1: Different representations of the BDS data recorded for bpyPDMS-NiBr2 at a few temperatures as 263 K, 283 K, 303 K, 323 K namely (a) real part of conductivity (σ'), (b) imaginary part of modulus (M''), (c) imaginary part of dielectric permittivity (ε''), (d) real part of dielectric permittivity (ε') vs. frequency.

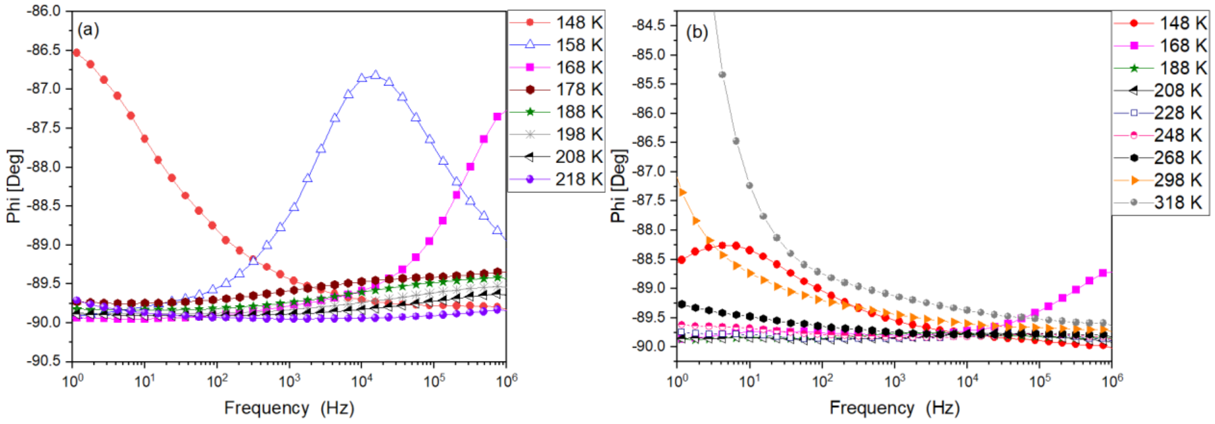

Figure S2: Representation of phase angle (Phi) at selected temperatures for (a) neat PDMS and (b) bpyPDMS-$ZnCl_2$ as a function of frequency.

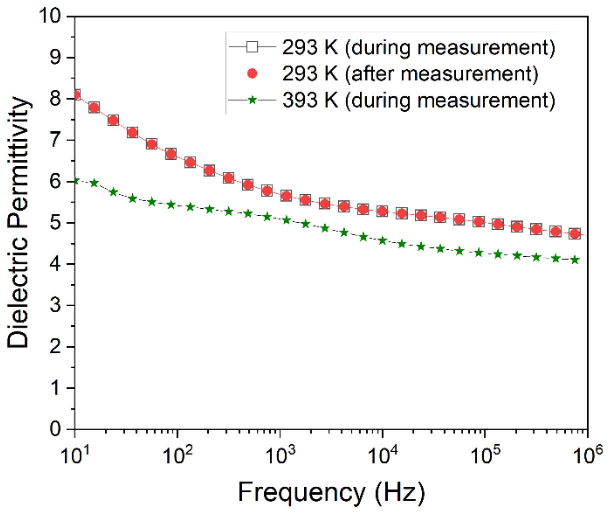

Figure S3: Frequency dependence of real part of dielectric permittivity for bpyPDMS-$NiBr_2$ at 293 K (during measurement), at 293 K (after measurement) and, at 393 K (during measurement).

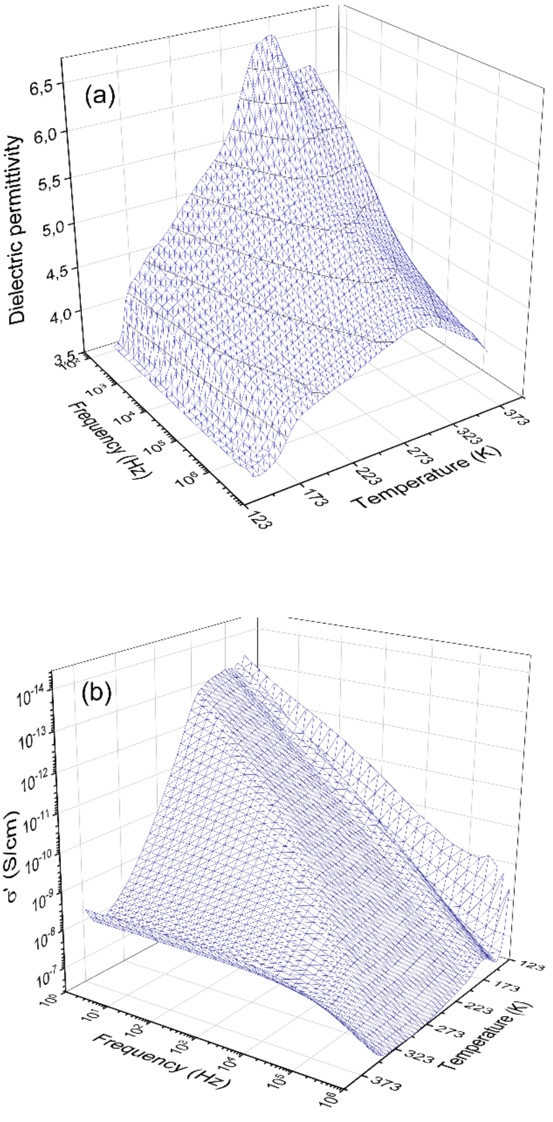

Figure S4: Frequency-temperature dependences of (a) real part of dielectric permittivity (b) real part of conductivity (σ’) for bpyPDMS-$NiBr_2$ sample.
